# The renal urate transporter SLC17A1 locus: confirmation of association with gout

**DOI:** 10.1186/ar3816

**Published:** 2012-04-27

**Authors:** Jade E Hollis-Moffatt, Amanda J Phipps-Green, Brett Chapman, Gregory T Jones, Andre van Rij, Peter J Gow, Andrew A Harrison, John Highton, Peter B Jones, Grant W Montgomery, Lisa K Stamp, Nicola Dalbeth, Tony R Merriman

**Affiliations:** 1 Department of Biochemistry, University of Otago, 710 Cumberland Street, Dunedin 9054, New Zealand; 2 Queensland Institute of Medical Research, 300 Herston Road, 4006 Brisbane, Queensland, Australia; 3 Department of Medicine, University of Otago, 210 Great King Street, Dunedin 9016, New Zealand; 4 Department of Rheumatology, Middlemore Hospital, 100 Hospital Road, Auckland 2025, New Zealand; 5 Department of Medicine, University of Otago, 23A Mein Street, Wellington 6242, New Zealand; 6 Department of Medicine, University of Auckland, 2 Park Road, Auckland 1023, New Zealand; 7 Department of Medicine, University of Otago, 2 Riccarton Avenue, Christchurch 8140, New Zealand

## Abstract

**Introduction:**

Two major gout-causing genes have been identified, the urate transport genes *SLC2A9 *and *ABCG2*. Variation within the *SLC17A1 *locus, which encodes sodium-dependent phosphate transporter 1, a renal transporter of uric acid, has also been associated with serum urate concentration. However, evidence for association with gout is equivocal. We investigated the association of the *SLC17A1 *locus with gout in New Zealand sample sets.

**Methods:**

Five variants (*rs1165196, rs1183201, rs9358890, rs3799344, rs12664474*) were genotyped across a New Zealand sample set totaling 971 cases and 1,742 controls. Cases were ascertained according to American Rheumatism Association criteria. Two population groups were studied: Caucasian and Polynesian.

**Results:**

At *rs1183201 *(*SLC17A1*), evidence for association with gout was observed in both the Caucasian (odds ratio (OR) = 0.67, *P *= 3.0 × 10^-6^) and Polynesian (OR = 0.74, *P *= 3.0 × 10^-3^) groups. Meta-analysis confirmed association of *rs1183201 *with gout at a genome-wide level of significance (OR = 0.70, *P *= 3.0 × 10^-8^). Haplotype analysis suggested the presence of a common protective haplotype.

**Conclusion:**

We confirm the *SLC17A1 *locus as the third associated with gout at a genome-wide level of significance.

## Introduction

Regulation of serum urate concentration is central to the development of gout, with renal uric acid excretion a critical checkpoint [1]. Genome-wide association scans examining the genetic control of serum urate concentrations have identified two renal urate transporters - *SLC2A9 *and *ABCG2 *- that have a strong effect on gout risk in multiple ethnic groups [2]. Whilst other loci (*SLC22A11, GCKR, INHBC, SLC17A1, RREB1, PDZK1, SLC16A9, LRRC16A*) have been associated with serum urate concentrations at a genome-wide level of significance in genome-wide association scans [3,4], only some of them (*SLC22A11, GCKR, INHBC, SLC17A1*) were associated with gout at a nominal level of significance (*P *< 0.05) in 1,100 cases nested within a large genome-wide association scan population-based cohort [4]. To understand why some loci do not associate with gout, and to assess the weakly associated loci in clinical gout, it will be necessary to minimize heterogeneity owing to the type of gout (primary or secondary to other causes such as diuretic use) and to test for association in clinically proven cases.

The solute carrier family 17 member 1 (encoded by *SLC17A1*), also known as sodium phosphate transport protein 1 (NPT1), is expressed on the apical membrane of renal tubular cells and mediates sodium and inorganic phosphate co-transport [5]. Sodium-dependent transporter 1 has also been identified as a urate transport protein [6,7], probably secretory [7] with the gout-protective allele of I269T [8] leading to increased sodium-dependent transporter 1 activity [6] and, presumably, increased secretion of uric acid. Genome-wide association scans have shown that genetic variants associate with serum urate concentration in a Caucasian sample [3,4]. *SLC17A1 *has been associated with gout in a Japanese sample set (I269T (*rs1165196*), odds ratio (OR) = 0.55, *P *= 0.005) [8] but with conflicting results in Caucasian sample sets. Marker *rs1165205 *in *SLC17A3 *was first associated with gout (OR = 0.85, *P *= 0.002) [9]. A later study incorporating the same clinical material with additional cases and controls, however, reported reduced combined evidence for association with gout using a strongly correlated marker within *SLC17A1 *(*rs1165196, r*^2 ^= 0.96; OR = 0.89, *P *= 0.013) [4] - in this study the markers most strongly associated with serum urate were within *SLC17A1 *(*rs1165196 *and other tightly correlated markers), suggesting that this gene was more likely than *SLC17A3 *to harbor an etiological variant. A separate study reported no evidence in Caucasian for association with gout (*rs1183201, r*^2 ^with *rs1165196 *= 0.87, OR = 0.97, *P *= 0.68) [10]. This equivocal evidence for association with gout in a Caucasian population is notable given the genome-wide evidence for association with serum urate concentration [4]. Both studies had adequate power to detect association of a moderate effect size, but neither study used clinical criteria to define gout.

Here, we aimed to test the SLC17A1 locus for association with gout, in multiple ancestral groups, using cases defined as a diagnosis of gout by the 1977 American College of Rheumatology (ARA) clinical criteria. The variants tested were *rs1183201*, demonstrated to influence serum urate concentration in Caucasian populations [3], the maximally gout-associated SNP (*rs1165196 *(I269T)) in Japanese [8], and three other SNPs predicted to tag major variation in Polynesian populations.

## Materials and methods

### Study participants

There were a total of four New Zealand (NZ) case-control sample sets, one of Caucasian ancestry and three of different Polynesian ancestries (see Supplemental Table S1 in Additional file 1). The sample sets were Eastern Polynesian (EP; NZ Māori and Cook Islands, 284 cases and 349 controls), Western Polynesian (WP; Samoa, Tonga, Niue and Tokelau, 251 cases and 144 controls), combined Eastern and Western Polynesian (EP/WP; 15 cases and 21 controls) and Caucasian (421 cases and 1,228 controls; of the controls, 590 had been SNP typed genome wide [11,12]). The EP samples were further subdivided into two groups to remove effects of stratification, as described in more detail below, based on the estimated proportion of EP ancestry (EP/N, 236 cases and 192 controls; and EP/Z, 48 cases and 157 controls). All gout cases recruited had a diagnosis of gout confirmed according to the ARA preliminary diagnostic criteria [13]. Controls self-reported as having no history of gout. Recruitment of cases was approved by the NZ Multi-Region Ethics Committee (MEC/05/10/130), and recruitment of controls by the Lower South Ethics Committee. All patients provided written informed consent for the collection of samples and subsequent analysis.

Analysis of genome-wide microsatellite data indicates a difference in population structure between Samoa and NZ Māori [14] - with a Māori sample set estimated to be ~85% Polynesian and ~15% Caucasian ancestry, and a Samoan sample set estimated at ~70% Polynesian, ~15% Asian and smaller components of Melanesian and Caucasian ancestry [14]. Analysis of genome-wide SNP data by principal component analysis also shows a difference in the first component between Samoan and Cook Island genomes [15]. Given data also showing heterogeneity in association of *ABCG2 *with gout in EP and WP sample sets [16], the analysis groups here were EP and WP. People of combined EP and WP ancestry were included as a separate group.

### Power

The individual sample sets were inadequately powered to detect an effect size in gout equivalent to that reported previously (OR = 1.12) [4], with the largest dataset (Caucasian) estimated to have 29% power at α = 0.05. However, at larger effect sizes the Caucasian sample set was better powered (63% power at OR = 1.2, 90% power at OR = 1.3 and 99% power at OR = 1.4). The smaller individual Polynesian sample sets had less power (for OR = 1.4: EP = 67% and WP = 45% using a Han Chinese Beijing (CHB) estimate of minor allele frequency = 0.18), although the combined Polynesian sample set was adequately powered with 88% power at OR = 1.4.

### SNP selection and determination of genotypes

**Figure 1:**
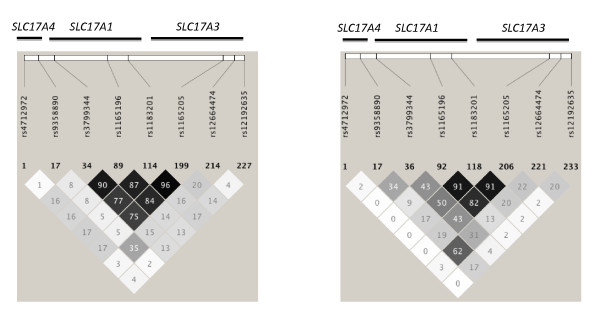
**Intermarker linkage disequilibrium for Caucasian and Chinese populations**. Intermarker linkage disequilibrium *r*^2 ^values for Caucasian (Centre d'Etude du Polymorphisme Humain; left) and Chinese (Han Chinese Beijing; right) populations. Approximate gene positions are shown. Diagram generated with Haploview using data from www.hapmap.org.

Using CHB HapMap data as the most closely related and available population to Polynesia, Haploview software (Broad Institute, Cambridge, MA, USA) was used to define four haplotype blocks using the Gabriel confidence interval method covering *SLC17A1 *(defined by *rs4712972 *(25.772 Mb) to *rs12192635 *(25.881 Mb)). Variants tagging major haplotypes were selected: *rs9358890 *in block 1, *rs3799344 *in block 2, *rs1183201 *in block 3 (previously associated with control of serum urate concentration) [3] and *rs12664474 *in block 4. The haplotype blocks extended into flanking genes (*SLC17A4 *and *SLC17A3*). In Centre d'Etude du Polymorphisme Humain (CEU) Caucasian population and the CHB population, *rs1183201 *and *rs3799344 *exhibited some intermarker linkage disequilibrium (LD) (*r*^2 ^= 0.77 and 0.50, respectively) and *rs9358890 *and *rs12664474 *also exhibited LD (*r*^2 ^= 0.35 and 0.62, respectively). SNP rs9358890 is in *SLC17A4*, and *rs12664474 *is in *SLC17A3*. SNP rs1165196 (*SLC17A1*) was also selected, in strong LD with *rs1183201 *(*r*^2 ^= 0.87 in CEU and 0.91 in CHB) (Figure 1).

Genotyping was done by TaqMan^® ^SNP genotyping assays (Applied Biosystems, Foster City, CA, USA) using a Lightcycler^® ^480 Real-Time PCR System (Roche, Indianapolis, IN, USA) for four SNPs: *rs1183201 *(assay ID: C_1911034_10), *rs9358890 *(assay ID: C_25595118_10), *rs3799344 *(assay ID: C_194536_10) and *rs12664474 *(assay ID: C_11189653_10). SNP rs1165196 was genotyped using Sequenom technology (Sequenom, Inc. San Diego, CA, USA). SNPs *rs9358890 *and *rs12664474 *had been genotyped over 590 of the Caucasian controls on the Affymetrix 6 SNP array (Affymetrix, Santa Clara, CA, USA) [12] - genotypes were imputed for *rs3799344 *and *rs1183201*with IMPUTE2, using HapMap3 CEU (NCBI Build 36 (db126b)) as reference haplotypes.

### Statistical analysis

ORs were calculated using PLINK software [17]. Because the case-control sample sets were not matched for sex, association analysis also included sex as a possible confounder. Analysis of association of haplotypes was also performed using PLINK. Meta-analysis was carried out using Rmeta software (within STATA 8.0, Stata, College Station, TX, USA) to calculate the combined Mantel-Haenszel OR using a fixed-effects model and the Breslow-Day test for heterogeneity.

The Māori population of NZ is admixed, primarily Caucasian. This leads to genetic stratification, which is a confounding factor for case-control genetic studies, especially when the prevalence of disease differs between the interbreeding populations. The prevalence of gout in NZ Māori is approximately double that in NZ Caucasian [18]. Given this, it is not surprising that the EP case sample set, which is predominantly NZ Māori (78% of cases, 93% of controls), has a significantly greater proportion of self-reported EP grandparents than does the control sample set (average of 3.1 EP grandparents in cases vs. 2.5 in controls, *P *= 3.1 × 10^-14 ^by *t *test). Sixty-seven biallelic genomic control markers (see Supplemental Table 2 in Additional file 1) were genotyped in the EP sample set, and STRUCTURE software [19] was used to estimate the individual proportion of EP ancestry. This estimation was performed using the following parameters: number of populations assumed to be two, 30,000 burn-in period, and 100,000 Markov chain Monte Carlo replications after burn-in. Caucasian control individuals genotyped for the 67 markers were included as representative of the ancestral Caucasian population to aid in population clustering, although we were unable to include EP ancestral representatives. Plots of self-reported ancestry versus STRUCTURE estimated ancestry are shown in Supplemental Figure 1 in Additional file 1. For association analysis we created two datasets matched for EP ancestry - EP/N with estimated EP ancestry > 0.65 (the geometric mean; 236 cases and 192 controls), and EP/Z with estimated EP ancestry ≤ 0.65 (48 cases and 157 controls). The estimated average proportion of EP ancestry in the EP/N sample set was 0.90 in cases and 0.88 in controls, and for the EP/Z group was 0.41 in cases and 0.40 in controls.

## Results

Association with gout was observed in the NZ Caucasian sample set for *rs1165196, rs1183201, rs3799344 *and *rs12664474 *(OR = 0.71 (95% confidence interval (CI) = 0.60 to 0.83), *P *= 5.5 × 10^-5^; OR = 0.67 (95% CI = 0.57 to 0.79), *P *= 3.0 × 10^-6^; OR = 0.69 (95% CI = 0.58 to 0.81), *P *= 2.8 × 10^-5^; and OR = 1.36 (95% CI = 1.12 to 1.66), *P *= 1.3 × 10^-3^, respectively), but not for *rs9358890 *(OR = 1.31 (95% CI = 0.93 to 1.85), *P *= 0.17) (Table 1). Given the low LD between *rs12664474 *and *rs1183201 *in CEU (*r*^2 ^= 0.16), suggesting the possibility of an independent effect at *rs12664474*, we tested for association of *rs12664474 *conditional on genotype at *rs1183201 *in the NZ Caucasian samples; there was no evidence for a separate genetic effect on gout risk at *rs12664474 *(*P *= 0.37). We also tested for conditional associations at *rs1183201 *and *rs1165196 *(*r*^2 ^in controls = 0.90) - there was association at *rs1183201 *conditional on genotype at *rs1165196 *(*P *= 0.007), but not at *rs1165196 *when conditioned on genotype at *rs1183201 *(*P *= 0.14).

**Table 1:** Association analysis in New Zealand case-control sample sets

**SNP**^a^	Cases			MAF	Controls			MAF	*P*_unadjusted_	*P*_adjusted_^b^	OR (95% CI)	HWE case	HWE control
***rs1165196***	**TT**	**CT**	**CC**	**C**	**TT**	**CT**	**CC**	**C**					
Caucasian	167 (0.400)	201 (0.482)	49 (0.118)	0.359	389 (0.318)	590 (0.482)	246 (0.200)	0.442	1.0 × 10^-4^	5.5 × 10^-5^	0.71 (0.60 to 0.83)	0.33	0.41
EP/N	130 (0.570)	86 (0.377)	12 (0.053)	0.241	98 (0.516)	77 (0.405)	15 (0.079)	0.282	0.19	0.26	0.81 (0.60 to 1.11)	0.65	0.98
EP/Z	16 (0.364)	22 (0.500)	6 (0.136)	0.386	53 (0.358)	64 (0.432)	31 (0.209)	0.426	0.51	0.88	0.85 (0.52 to 1.38)	0.72	0.16
WP	138 (0.556)	99 (0.399)	11 (0.044)	0.244	69 (0.496)	54 (0.388)	16 (0.115)	0.309	0.05	0.06	0.72 (0.52 to 1.00)	0.20	0.28
EP/WP	10 (0.667)	5 (0.333)	0 (0.000)	0.167	6 (0.316)	10 (0.526)	3 (0.158)	0.421	0.02	0.07	0.28 (0.09 to 0.87)	0.33	0.73
Combined^c^									5.7 × 10^-7^		0.72 (0.64 to 0.82)		
*rs1183201*^d^	TT	TA	AA	A	TT	TA	AA	A					
Caucasian	158 (0.384)	205 (0.499)	48 (0.117)	0.366	356 (0.291)	608 (0.496)	261 (0.213)	0.461	2.0 × 10^-6^	3.0 × 10^-6^	0.67 (0.57 to 0.79)	0.13	0.89
EP/N	130 (0.570)	86 (0.377)	12 (0.053)	0.241	96 (0.505)	78 (0.411)	16 (0.084)	0.289	0.11	0.14	0.78 (0.57 to 1.06)	0.65	0.98
EP/Z	15 (0.349)	21 (0.488)	7 (0.163)	0.407	52 (0.349)	61 (0.409)	36 (0.242)	0.446	0.49	0.95	0.85 (0.52 to 1.39)	0.94	0.04
WP	132 (0.543)	101 (0.416)	10 (0.041)	0.249	68 (0.479)	57 (0.401)	17 (0.120)	0.320	0.03	0.03	0.70 (0.51 to 0.97)	0.08	0.35
EP/WP	9 (0.600)	6 (0.400)	0 (0.000)	0.200	7 (0.350)	10 (0.500)	3 (0.150)	0.400	0.07	0.11	0.38 (0.13 to 1.12)	0.25	0.99
Combined^c^									3.0 × 10^-8^		0.70 (0.62 to 0.79)		
*rs9358890 *	AA	AG	GG	G	AA	AG	GG	G					
Caucasian	366 (0.884)	47 (0.114)	1 (0.002)	0.059	1114 (0.912)	104 (0.085)	4 (0.003)	0.046	0.12	0.17	1.31 (0.93 to 1.85)	0.69	0.35
EP/N	97 (0.418)	99 (0.427)	36 (0.155)	0.369	80 (0.419)	78 (0.408)	33 (0.173)	0.377	0.80	0.71	0.96 (0.73 to 1.28)	0.21	0.07
EP/Z	34 (0.753)	11 (0.244)	0 (0.000)	0.122	129 (0.827)	26 (0.167)	1 (0.006)	0.090	0.44	0.42	1.41 (0.67 to 2.96)	0.35	0.80
WP	110 (0.440)	101 (0.404)	39 (0.156)	0.358	73 (0.507)	61 (0.424)	10 (0.069)	0.281	0.03	0.06	1.43 (1.04 to 1.95)	0.06	0.57
EP/WP	8 (0.533)	7 (0.467)	0 (0.000)	0.233	12 (0.571)	7 (0.333)	2 (0.095)	0.262	0.78	0.87	0.86 (0.29 to 2.55)	0.24	0.53
Combined^c^									0.05		1.19 (1.00 to 1.41)		
*rs3799344 *	CC	CT	TT	T	CC	CT	TT	T					
Caucasian	165 (0.404)	193 (0.473)	50 (0.123)	0.359	379 (0.309)	592 (0.483)	255 (0.207)	0.450	5.9 × 10^-6^	2.8 × 10^-5^	0.69 (0.58 to 0.81)	0.58	0.38
EP/N	128 (0.561)	88 (0.386)	12 (0.053)	0.246	97 (0.524)	73 (0.395)	15 (0.081)	0.278	0.46	0.37	0.84 (0.62 to 1.15)	0.53	0.80
EP/Z	17 (0.378)	22 (0.489)	6 (0.133)	0.378	53 (0.353)	64 (0.427)	33 (0.220)	0.433	0.35	0.72	0.79 (0.49 to 1.29)	0.79	0.49
WP	136 (0.551)	98 (0.397)	13 (0.053)	0.251	64 (0.457)	59 (0.421)	17 (0.121)	0.332	0.02	0.02	0.67 (0.49 to 0.93)	0.39	0.55
EP/WP	10 (0.667)	5 (0.333)	0 (0.000)	0.167	7 (0.350)	10 (0.500)	3 (0.150)	0.400	0.04	0.08	0.30 (0.10 to 0.95)	0.44	0.85
Combined^c^									7.4 × 10^-8^		0.71 (0.62 to 0.80)		
*rs12664474 *	AA	AG	GG	G	AA	AG	GG	G					
Caucasian	264 (0.632)	134 (0.321)	20 (0.048)	0.208	867 (0.707)	321 (0.262)	38 (0.031)	0.162	0.01	1.2 × 10^-3^	1.36 (1.12 to 1.66)	0.57	0.22
EP/N	90 (0.383)	104 (0.443)	41 (0.174)	0.396	74 (0.392)	77 (0.407)	38 (0.201)	0.405	0.79	0.17	0.96 (0.73 to 1.27)	0.25	0.03
EP/Z	25 (0.543)	18 (0.391)	3 (0.065)	0.261	107 (0.704)	39 (0.257)	6 (0.039)	0.168	0.05	0.13	1.75 (1.00 to 3.05)	0.92	0.32
WP	102 (0.411)	106 (0.427)	40 (0.161)	0.375	63 (0.444)	67 (0.472)	12 (0.085)	0.320	0.13	0.10	1.27 (0.93 to 1.73)	0.16	0.32
EP/WP	7 (0.467)	6 (0.400)	2 (0.133)	0.333	12 (0.571)	7 (0.333)	2 (0.095)	0.262	0.51	0.38	1.41 (0.51 to 3.92)	0.70	0.53
Combined^c^									2.0 × 10^-3^		1.25 (1.09 to 1.43)		

CI, confidence interval; HWE, **{AU Query: Define}**; MAF, {**AU Query: Define}**; OR, odds ratio. ^a^Genotyping success rate: *rs1165196 *Caucasian, Eastern Polynesian (EP), Western Polynesian (WP) and EP/WP - cases 99.0%, 95.8%, 98.8% and 100%, respectively, controls 99.8%, 96.8%, 96.5% and 90.5%, respectively; *rs1183201 *Caucasian, EP, WP and EP/WP - cases 97.6%, 95.4%, 96.8% and 100%, respectively, controls 99.7%, 97.1%, 98.6% and 95.2%, respectively; *rs9358890 *Caucasian, EP, WP and EP/WP - cases 98.3%, 97.5%, 99.6% and 100%, respectively, controls 99.2%, 99.4%, 100% and 90.5%, respectively; *rs3799344 *Caucasian, EP, WP, EP/WP - cases 96.9%, 96.1%, 98.4% and 100%, respectively, controls 99.8%, 96.0%, 97.2% and 95.2%, respectively; *rs12664474 *Caucasian, EP, WP, EP/WP - cases 99.3%, 98.9%, 98.8% and 100%, respectively, controls 99.8%, 97.7%, 98.6% and 100%, respectively. ^b^Adjusted for sex. ^c^Meta-analysis by the Mantel-Haenszel method. Breslow-Day test for heterogeneity: *rs1165196, P *= 0.45; *rs1183201, P *= 0.75; rs9358890, *P *= 0.77; *rs3799344, P *= 0.58; *rs12664474, P *= 0.97. ^d^Population attributable risk (the reduction in gout incidence if the risk variant was absent) for positivity of the risk (T) allele of *rs1183201 *is 38.2% for Caucasian, 21.4% for EP/N, 27.3% for EP/N and 41.3% for WP.

**Table 2:** Association of four-marker *rs9358890-rs3799344-rs1183201-rs12664474 *haplotypes with gout

	Frequency			
			
Haplotype^a^	Case	Control	OR (95% CI)	*P *value
Caucasian				
A-T-A-A	270 (0.339)	1044 (0.428)	0.66 (0.56 to 0.78)	1.5 × 10^-6^
A-C-T-A	327 (0.410)	876 (0.360)	1.22 (1.03 to 1.44)	0.014
A-C-T-G	121 (0.152)	284 (0.117)	1.34 (1.06 to 1.69)	0.015
G-C-T-G	47 (0.059)	104 (0.043)	1.39 (0.98 to 1.98)	0.067
EP/N				
A-T-A-A	107 (0.239)	101 (0.274)	0.84 (0.61 to 1.15)	0.27
A-C-T-A	160 (0.356)	109 (0.297)	1.33 (0.99 to 1.78)	0.06
G-C-T-G	161 (0.360)	140 (0.380)	0.93 (0.70 to 1.23)	0.60
EP/Z				
A-T-A-A	32 (0.372)	120 (0.414)	0.84 (0.51 to 1.38)	0.49
A-C-T-A	32 (0.372)	111 (0.384)	0.95 (0.58 to 1.56)	0.84
G-C-T-G	9 (0.105)	26 (0.090)	1.19 (0.53 to 2.64)	0.68
A-C-T-G	10 (0.116)	22 (0.075)	1.63 (0.74 to 3.59)	0.23
WP				
A-C-T-A	171 (0.357)	96 (0.343)	1.07 (0.78 to 1.45)	0.70
A-T-A-A	116 (0.241)	89 (0.318)	0.68 (0.49 to 0.94)	0.021
G-C-T-G	171 (0.356)	77 (0.275)	1.46 (1.06 to 2.02)	0.021
A-C-T-G	12 (0.025)	12 (0.043)	0.57 (0.25 to 1.29)	0.17
EP/WP				
A-T-A-A	5 (0.167)	16 (0.400)	0.30 (0.10 to 0.95)	0.035
A-C-T-A	15 (0.500)	13 (0.324)	2.08 (0.78 to 5.50)	0.14
G-C-T-G	6 (0.200)	11 (0.275)	0.66 (0.21 to 2.05)	0.47

CI, confidence interval; EP, Eastern Polynesian; OR, odds ratio; WP, Western Polynesian. ^a^Haplotypes are listed in descending order of control frequency; those with frequency < 0.03 were excluded.

The five variants were then tested for association in the Polynesian sample sets (Table 1), with the only evidence for association in individual sample sets coming from WP at *rs1183201 *(OR = 0.70, *P *= 0.03) and *rs3799344 *(OR = 0.67, *P *= 0.02). However, meta-analysis of the Polynesian sample sets - carried out to increase power - replicated the association observed in Caucasian at *rs1165196 *(OR = 0.75 (95% CI = 0.60 to 0.94), *P *= 0.013, *P*_Het _= 0.33), *rs1183201 *(OR = 0.74 (95% CI = 0.61 to 0.91), *P *= 0.003, *P*_Het _= 0.57) and *rs3799344 *(OR = 0.74 (95% CI = 0.61 to 0.90), *P *= 0.003, *P*_Het _= 0.33), but not at *rs9358890 *(OR = 1.15 (95% CI = 0.95 to 1.40), *P *= 0.16, *P*_Het _= 0.28) or *rs12664474 *(OR = 1.16 (95% CI = 0.96 to 1.40), *P *= 0.13, *P*_Het _= 0.23).

The Caucasian and Polynesian sample sets were combined in meta-analysis for *rs1165196 *(OR = 0.72 (95% CI = 0.64 to 0.82), *P *= 5.7 × 10^-7^), *rs1183201 *(OR = 0.70 (95% CI = 0.62 to 0.79), *P = *3.0 × 10^-8^, *P*_Het _= 0.64), *rs9358890 *(OR = 1.19 (95% CI = 1.00 to 1.41), *P = *0.05, *P*_Het _= 0.37), *rs3799344 *(OR = 0.71 (95% CI = 0.62 to 0.80), *P = *7.4 × 10^-8^, *P*_Het _= 0.43), and *rs12664474 *(OR = 1.25 (95% CI = 1.09 to 1.43), *P = *2.0 × 10^-3^, *P*_Het _= 0.23). Of the five SNPs, *rs1183201 *was the only one significant at a genome-wide level of significance (*P *< 5 × 10^-8^). None of the SNPs were significantly associated with serum urate in either the Caucasian controls (for whom there were serum urate data available; see Supplemental Table 1 in Additional file 1) or the less admixed combined WP and EP/N controls (all *P *> 0.28).

Because haplotypes are multi-allelic we analyzed association of haplotypes with gout, with the purpose of investigating the mechanism of effect - that is, whether risk and/or protective variants are present and comparing association pattern between populations. Analysis of four-marker haplotypes (*rs9358890*-*rs3799344*-*rs1183201*-*rs12664474*; Table 2) revealed the most consistent evidence for association to come from the A-T-A-A haplotype (OR = 0.30 to 0.84), with significant association in the Caucasian, WP and EP/WP sample sets (*P *= 1.5 × 10^-6 ^to 0.035).

## Discussion

Genetic regulators of serum urate concentration that have been previously associated with gout at a genome-wide level of significance (*P *< 5 × 10^-8^) in Caucasian samples are *SLC2A9 *[4,9,20] and *ABCG2 *[4,9,16]. Here, we provide strong evidence for a role of the *SLC17A1 *locus in gout in a Caucasian population (*rs1183201*, OR = 0.67, *P *= 3.0 × 10^-6^; Table 1) that was replicated in Polynesian samples, with the minor allele of *rs1183201 *also conferring a similar degree of risk (OR = 0.74, *P*_meta-analysis _= 3.0 × 10^-3^). The haplotype data (Table 2) are consistent with the presence of at least one genetic variant influencing the risk of gout at the *SLC17A1 *locus. We hypothesize that the variant is protective of gout and is contained on a common haplotype (27 to 43%; A-T-A-A), conferring significant protection in three out of the five sample sets (also with OR < 1 in both EP sample sets). There were no haplotypes consistently conferring risk. Combining the populations provided a genome-wide level of significance for association of *rs1183201 *with gout (OR = 0.70, *P *= 3.0 × 10^-8^). This confirms the *SLC17A1 *locus as the third associated with gout.

The role of *SLC17A1 *has been previously evaluated in gout in a Japanese sample set [8], with the nonsynonymous variant I269T (*rs1165196*) having the strongest evidence for association (OR = 0.55, *P *= 0.004, minor allele (269T) protective). *rs1165196 *is in strong LD with *rs1183201 *-the maximally associated variant in our study - in Japanese (HapMap JPT) and Caucasian (HapMap CEU) samples (*r*^2 ^= 0.92 and *r*^2 ^= 0.87, respectively). Given that I269T has been shown to affect the function of SLC17A1, with the protective variant (269T, minor allele of *rs1165196*) leading to increased activity in *Xenopus *oocytes and, presumably, increased renal elimination of urate [6], it is therefore possible that *rs1165196 *is an etiological variant. However, we found no evidence in the Caucasian sample set supporting association at *rs1165196 *when conditioned on genotype at *rs1183201*, and association was weaker at *rs1165196 *than *rs1183201 *in combined Caucasian and Polynesian meta-analysis (OR = 0.72, *P *= 5.7 × 10^-7 ^and OR = 0.70, *P *= 3 × 10^-8^, respectively) and in Polynesian alone (OR = 0.75, *P *= 0.013 and OR = 0.74, *P *= 0.003, respectively) (we did not conditionally analyze the small Polynesian sample sets). Ostensibly this observation argues that *rs1183201 *(or a variant in strong LD) is more likely than *rs1165196 *to be an etiological variant within *SLC17A1*. Given that *rs1165196 *has a stronger effect in serum urate levels in Caucasian ([4] β = 6.205 vs. 6.050 for *rs1183201*) populations, however, this interpretation should await further testing in larger gout and serum urate sample sets.

In the Caucasian analysis, *rs1183201 *was strongly associated with gout (OR = 0.67 (95% CI = 0.57 to 0.79)). This SNP, or SNPs in strong LD, has been studied for association with gout in two previous studies: Yang and colleagues [4], with OR = 0.89 (95% CI = 0.82 to 0.98); and Stark and colleagues [10], with OR = 0.97 (95% CI = 0.86 to 1.11). The strength of effect in our study is considerably greater than the previous studies, with a 95% CI that does not overlap with either study. Given that the control allele frequencies were similar between our study and those of Yang and colleagues [4] and Stark and colleagues [10] (0.461 (*rs1183201*), 0.46 (*rs1165196*), and 0.487 (*rs1183201*), respectively), the differences in effect size are therefore caused by differences in allele frequency in case sample sets. Differences in ascertainment of cases are a possible reason for this effect. Here, cases were clinically ascertained by ARA criteria with exclusion of patients suspected of having diuretic-induced gout. In Yang and colleagues' study, cases were drawn from five population-based cohorts and were ascertained by: self-report or allopurinol treatment (AGES Reykjavik Study); self-report (Atherosclerosis Risk in Communities Study); receiving gout medication (allopurinol, colchicine, probenecid; Cardiovascular Health Study); self-report (Framingham Heart Study); and receiving gout medication (allopurinol, colchicine, probenecid, benzbromarone; Rotterdam Study) [4]. In Stark and colleagues' study, cases were ascertained by self-report and review of medical history [10].

In the study by Yang and colleagues no details were included about the inclusion, or otherwise, of diuretic-induced cases [4]; and in the study by Stark and colleagues 36.1% of cases were taking diuretic medication [10]. The use of self-reported gout probably results in participants without clinical evidence for gout being included in case sample sets; for example, only 69% of men who self-reported as new cases of gout met the ARA classification criteria for gout [21], and reliability and sensitivity for self-reported gout have been estimated at 63 to 73% and 84%, respectively [22]. Although the reliability of use of medications such as allopurinol, colchicine, probenecid and benzbromarone has not been extensively investigated for gout classification, the use of allopurinol prescription gives a positive predictive value of 39% for probable/definite gout [23]. Certainly, gout case sample sets ascertained using such indirect criteria had lower effect sizes reported at *SLC2A9*, compared with sets using ARA criteria [20]. The method of ascertainment in the previous studies [4,10] would thus reduce power to detect association at *SLC17A1 *owing to inclusion of nongout participants in the case sample sets. The use of diuretic medications is well established as a gout risk factor [24], perhaps by inhibition of urate excretion mediated by human organic anion transporter 4 [25]. In Stark and colleagues' study [10], this could reduce power to detect association by studying cases with secondary gout, since the inhibitory effect of diuretic medication on organic anion transporter 4-mediated renal urate excretion would predominate over the genetic effect on gout risk mediated by the *SLC17A1 *locus. It is also conceivable that diuretics directly influence the function of urate transporters encoded in the locus. The loop diuretic bumetanide has recently been shown to be a transport substrate for sodium-dependent transporter 4 (encoded by *SLC17A3*), and functional polymorphic variants are likely to influence transport ability [26]. Given the likelihood that gene-diuretic interactions exist, one would be prudent to exclude gout cases taking diuretic medication as a potential confounding factor in order to evaluate the direct effect of genetic variation in the *SLC17A1 *locus on primary gout.

## Conclusion

We provide, for the first time, a genome-wide level of evidence supporting a role for genetic variation in the *SLC17A1 *locus in the etiology of gout. This is the third urate transport locus associated with gout with this robust level of evidence, and our results further emphasize the importance of urate transport in gout.

## Abbreviations

ABC: ATP-binding cassette; ARA: American Rheumatism Association; CEU: Centre d'Etude du Polymorphisme Humain; CHB: Han Chinese Beijing; CI: confidence interval; EP: Eastern Polynesian; LD: linkage disequilibrium; NZ: New Zealand; OR: odds ratio; SLC: solute carrier family; SNP: single nucleotide polymorphism; WP: Western Polynesian.

## Competing interests

The authors declare that they have no competing interests.

## Authors' contributions

JEH-M, AJP-G and TRM helped to design the study, oversee its execution, and prepare the manuscript. GTJ, AvR, PJG, AAH, JH, PBJ, LKS and ND helped to provide clinical recruitment and prepare the manuscript. BC and GWM helped to collect data and prepare the manuscript. All authors read and approved the final manuscript.

## Supplementary Material

Additional file 1 **Supplemental Table 1 presenting participant demographic and clinical details, Supplemental Table 2 presenting genomic control SNPs, and Supplemental Figure 1 showing the correlation of self-reported number of EP grandparents with estimated EP ancestry using 67 genomic control markers**. Click here for file

## References

[B1] Simmonds HA McBride MB Hatfield PJ Graham R McCaskey J Jackson M Polynesian women are also at risk for hyperuricaemia and gout because of a genetic defect in renal urate handling Br J Rheumatol 1994 33 932 937 7921753 10.1093/rheumatology/33.10.932

[B2] Merriman TR Dalbeth N The genetic basis of hyperuricaemia and gout Joint Bone Spine 2011 38 35 40 20472486 10.1016/j.jbspin.2010.02.027

[B3] Kolz M Johnson T Sanna S Teumer A Vitart V Perola M Mangino M Albrecht E Wallace C Farrall M Johansson A Nyholt DR Aulchenko Y Beckmann JS Bergmann S Bochud M Brown M Campbell H EUROSPAN Consortium Connell J Dominiczak A Homuth G Lamina C McCarthy MI ENGAGE Consortium Meitinger T Mooser V Munroe P Nauck M Peden J Meta-analysis of 28,141 individuals identifies common variants within five new loci that influence uric acid concentrations PLoS Genet 2009 5 e1000504 19503597 10.1371/journal.pgen.1000504 PMC2683940

[B4] Yang Q Kottgen A Dehghan A Smith AV Glazer NL Chen MH Chasman DI Aspelund T Eiriksdottir G Harris TB Launer L Nalls M Hernandez D Arking DE Boerwinkle E Grove ML Li M Linda Kao WH Chonchol M Haritunians T Li G Lumley T Psaty BM Shlipak M Hwang SJ Larson MG O'Donnell CJ Upadhyay A van Duijn CM Multiple genetic loci influence serum urate and their relationship with gout and cardiovascular disease risk factors Circ Cardiovasc Genet 2010 3 523 530 20884846 10.1161/CIRCGENETICS.109.934455 PMC3371395

[B5] Busch AE Schuster A Waldegger S Wagner CA Zempel G Broer S Biber J Murer H Lang F Expression of a renal type I sodium/phosphate transporter (NaPi-1) induces a conductance in Xenopus oocytes permeable for organic and inorganic anions Proc Natl Acad Sci USA 1996 93 5347 5351 8643577 10.1073/pnas.93.11.5347 PMC39248

[B6] Iharada M Miyaji T Fujimoto T Hiasa M Anzai N Omote H Moriyama Y Type 1 sodium-dependent phosphate transporter (SLC17A1 protein) is a Cl(-)-dependent urate exporter J Biol Chem 2010 285 26107 26113 20566650 10.1074/jbc.M110.122721 PMC2924012

[B7] Uchino H Tamai I Yamashita K Minemoto Y Sai Y Yabuuchi H Miyamoto K Takeda E Tsuji A p-aminohippuric acid transport at renal apical membrane mediated by human inorganic phosphate transporter NPT1 Biochem Biophys Res Commun 2000 270 254 259 10733936 10.1006/bbrc.2000.2407

[B8] Urano W Taniguchi A Anzai N Inoue E Kanai Y Yamanaka M Endou H Kamatani N Yamanaka H Sodium-dependent phosphate cotransporter type 1 sequence polymorphisms in male patients with gout Ann Rheum Dis 2010 69 1232 1234 19556210 10.1136/ard.2008.106856

[B9] Dehghan A Köttgen A Yang Q Hwang SJ Kao WL Rivadeneira F Boerwinkle E Levy D Hofman A Astor BC Benjamin EJ van Duijn CM Witteman JC Coresh J Fox CS Association of three genetic loci with uric acid concentration and risk of gout: a genome-wide association study Lancet 2008 372 1953 1961 18834626 10.1016/S0140-6736(08)61343-4 PMC2803340

[B10] Stark K Reinhard W Grassl M Erdmann J Schunkert H Illig T Hengstenberg C Common polymorphisms influencing serum uric acid levels contribute to susceptibility to gout, but not to coronary artery disease PLoS One 2009 4 e7729 19890391 10.1371/journal.pone.0007729 PMC2766838

[B11] Roberts RL Van Rij AM Phillips LV Young S McCormick SP Merriman TR Jones GT Interaction of the inflammasome genes CARD8 and NLRP3 in abdominal aortic aneurysms Atherosclerosis 2011 218 123 126 21621776 10.1016/j.atherosclerosis.2011.04.043

[B12] Gretarsdottir S Baas AF Thorleifsson G Holm H den Heijer M de Vries JP Kranendonk SE Zeebregts CJ van Sterkenburg SM Geelkerken RH van Rij AM Williams MJ Boll AP Kostic JP Jonasdottir A Jonasdottir A Walters GB Masson G Sulem P Saemundsdottir J Mouy M Magnusson KP Tromp G Elmore JR Sakalihasan N Limet R Defraigne JO Ferrell RE Ronkainen A Ruigrok YM Genome-wide association study identifies a sequence variant within the DAB2IP gene conferring susceptibility to abdominal aortic aneurysm Nat Genet 2010 42 692 697 20622881 10.1038/ng.622 PMC4157066

[B13] Wallace SL Robinson H Masi AT Decker JL McCarty DJ Yu TF Preliminary criteria for the classification of the acute arthritis of primary gout Arthritis Rheum 1977 20 895 900 856219 10.1002/art.1780200320

[B14] Friedlaender JS Friedlaender FR Reed FA Kidd KK Kidd JR Chambers GK Lea RA Loo JH Koki G Hodgson JA Merriwether DA Weber JL The genetic structure of Pacific Islanders PLoS Genet 2008 4 e19 18208337 10.1371/journal.pgen.0040019 PMC2211537

[B15] Wollstein A Lao O Becker C Brauer S Trent RJ Nürnberg P Stoneking M Kayser M Demographic history of Oceania inferred from genome-wide data Curr Biol 2010 20 1983 1992 21074440 10.1016/j.cub.2010.10.040

[B16] Phipps-Green AJ Hollis-Moffatt JE Dalbeth N Merriman ME Topless R Gow PJ Harrison AA Highton J Jones PB Stamp LK Merriman TR A strong role for the ABCG2 gene in susceptibility to gout in New Zealand Pacific Island and Caucasian, but not Maori, case and control sample sets Hum Mol Genet 2010 19 4813 4819 20858603 10.1093/hmg/ddq412

[B17] Purcell S Neale B Todd-Brown K Thomas L Ferreira MA Bender D Maller J Sklar P de Bakker PI Daly MJ Sham PC PLINK: a tool set for whole-genome association and population-based linkage analyses Am J Hum Genet 2007 81 559 575 17701901 10.1086/519795 PMC1950838

[B18] Winnard D Wright C Taylor WJ Jackson G Te Karu L Gow PJ Arroll B Thornley S Gribben B Dalbeth D National prevalence of gout derived from administrative health data in Aotearoa New Zealand Rheumatology 2012 51 901 909 22253023 10.1093/rheumatology/ker361

[B19] Pritchard JK Stephens M Donnelly P Inference of population structure using multilocus genotype data Genetics 2000 155 945 959 10835412 10.1093/genetics/155.2.945 PMC1461096

[B20] Hollis-Moffatt JE Xu X Dalbeth N Merriman ME Topless R Waddell C Gow PJ Harrison AA Highton J Jones PB Stamp LK Merriman TR Role of the urate transporter SLC2A9 gene in susceptibility to gout in New Zealand Maori, Pacific Island, and Caucasian case-control sample sets Arthritis Rheum 2009 60 3485 3492 19877038 10.1002/art.24938

[B21] Choi HK Atkinson K Karlson EW Willett W Curhan G Purine-rich foods, dairy and protein intake, and the risk of gout in men N Engl J Med 2004 350 1093 1103 15014182 10.1056/NEJMoa035700

[B22] McAdams MA Maynard JW Baer AN Köttgen A Clipp S Coresh J Gelber AC Reliability and sensitivity of the self-report of physician-diagnosed gout in the campaign against cancer and heart disease and the atherosclerosis risk in the community cohorts J Rheumatol 2011 38 135 141 21123328 10.3899/jrheum.100418 PMC3285109

[B23] Harrold LR Saag KG Yood RA Mikuls TR Andrade SE Fouayzi H Davis J Chan KA Raebel MA Von Worley A Platt R Validity of gout diagnoses in administrative data Arthritis Rheum 2007 57 103 108 17266097 10.1002/art.22474

[B24] Roddy E Doherty M Epidemiology of gout Arthritis Res Ther 2010 12 223 21205285 10.1186/ar3199 PMC3046529

[B25] Hagos Y Stein D Ugele B Burckhardt G Bahn A Human renal organic anion transporter 4 operates as an asymmetric urate transporter J Am Soc Nephrol 2007 18 430 439 17229912 10.1681/ASN.2006040415

[B26] Jutabha P Anzai N Kitamura K Taniguchi A Kaneko S Yan K Yamada H Shimada H Kimura T Katada T Fukutomi T Tomita K Urano W Yamanaka H Seki G Fujita T Moriyama Y Yamada A Uchida S Wempe MF Endou H Sakurai H Human sodium phosphate transporter 4 (hNPT4/SLC17A3) as a common renal secretory pathway for drugs and urate J Biol Chem 2010 285 35123 35123 20810651 10.1074/jbc.M110.121301 PMC2966126

