# Supplementary material for: The renal urate transporter SLC17A1 locus: confirmation of association with gout
Source: Arthritis Res Ther. 2012 Apr 27;14(2):R92. doi: 10.1186/ar3816 (PMC3446466; doi:10.1186/ar3816)
Supplement: Additional file 1 — Supplemental Table 1 presenting participant demographic and clinical details, Supplemental Table 2 presenting genomic control SNPs, and Supplemental Figure 1 showing the correlation of self-reported number of EP grandparents with estimated EP ancestry using 67 genomic control markers. [file ar3816-S1.DOCX]

**Supplemental Material for:**

**The renal urate transporter SLC17A1 locus: confirmation of association with gout**

Jade E Hollis-Moffatt, Amanda J Phipps-Green, Brett Chapman, Gregory T Jones, Andre van Rij, Peter J Gow, Andrew A Harrison, John Highton, Peter B Jones, Grant W Montgomery, Lisa K Stamp, Nicola Dalbeth, Tony R Merriman

**Supplemental Table 1** Participant demographic and clinical details

|  | **Gout Mixed EP/WP** | **Gout EP** | **Gout WP** | **Gout Cauca’n** | **Control Mixed EP/WP** | **Control EP** | **Control WP** | **Control Cauca’n^1^** |
| --- | --- | --- | --- | --- | --- | --- | --- | --- |
| % male | 91.3 | 75.9 | 93.6 | 86.6 | 53.3 | 32.7 | 56.9 | 42.1 |
| Mean ± 1SD grandparents of relevant ancestry | 3.33 ± 0.83 | 3.03 ± 1.05 | 3.72 ± 0.58 | 4.00 ± 0.00 | 2.98 ± 0.84 | 2.46 ± 1.22 | 3.49 ± 0.86 | 4.00 ± 0.00 |
| Serum urate at recruitment, mmol/L (mean, range) | 0.51  (0.41-0.67) | 0.42  (0.17-0.70) | 0.45  (0.19-0.76) | 0.39  (0.13-0.69) | 0.41 (0.22-0.67) | 0.34 (0.15-0.58) | 0.36 (0.25-0.57) | 0.37 (0.13-0.52) |
| Age of onset, years (mean, range) | 29.9  (19-57) | 39.4  (14-74) | 34.4  (14-80) | 46.4  (10-83) | - | - | - | - |
| Age of recruitment, years (mean, range) | - | - | - | - | 33  (18-80) | 44  (17-85) | 38  (17-86) | 52  (17-95) |
| Mean number of gout attacks in past year | 7.8 | 10.4 | 13 | 6.5 | - | - | - | - |
| % first-degree relative with gout | 61.9 | 61.1 | 53.5 | 42.5 | 23.1 | 31.4 | 23 | 13.6 |
| Allopurinol treatment, % | 76.2 | 78.7 | 80.1 | 74.2 | - | - | - | - |
| Probenecid treatment, % | 5.3 | 6.1 | 10.5 | 5.6 | - | - | - | - |
| Body mass index  (mean, range) | 38.2  (27-38) | 35.1  (22-66) | 37.3  (22-93) | 30.4  (19-62) | 34.3 (24-42) | 32  (20-77) | 34.7  (19-77) | 27.8  (19-56) |
| Other conditions, % |  |  |  |  |  |  |  |  |
| Type 2 diabetes | 23.8 | 27.5 | 15.9 | 15.1 | 6.7 | 9.5 | 12.0 | 6.6 |
| Hypertension | 55.0 | 62.2 | 44.5 | 48.0 | 20.0 | 16.3 | 17.5 | 13.8 |
| Dyslipidaemia | 55.0 | 57.2 | 53.4 | 48.7 | 14.3 | 12.1 | 12.2 | 15.6 |
| Cardiovascular disease | 20.0 | 35.1 | 15.5 | 36.4 | 0 | 3.2 | 8.0 | 6.1 |
| Renal disease | 30.0 | 28.4 | 16.7 | 20.0 | 0 | 1.6 | 1.0 | 2.2 |

1 Clinical data are from 133 of the Caucasian (those not genome-wide genotyped) sample set.

**Supplemental Table 2** Genomic control SNPs

| **Taqman** | **Sequenom** | **PCR-RFLP^1^** |
| --- | --- | --- |
| *rs1183201* | *rs10025373* | *rs7725* |
| *rs9358890* | *rs1143634* | *rs573816* |
| *rs3799344* | *rs11536879* |  |
| *rs12664474* | *rs1205* |  |
| *rs2075876* | *rs2812378* |  |
| *rs1816532* | *rs3014875* |  |
| *rs13419122* | *rs344542* |  |
| *rs12401573* | *rs40401* |  |
| *rs6945435* | *rs452204* |  |
| *rs743777* | *rs4780884* |  |
| *rs10511216* | *rs4781011* |  |
| *rs12745968* | *rs4804221* |  |
| *rs1539438* | *rs4845622* |  |
| *rs729749* | *rs4889640* |  |
| *rs3738919* | *rs507879* |  |
| *rs1130214* | *rs6005863* |  |
| *rs755622* | *rs6819597* |  |
| *rs7901695* | *rs6835636* |  |
| *rs7578597* | *rs7811892* |  |
| *rs2043211* | *rs7842* |  |
| *rs10733113* | *rs795467* |  |
| *rs900865* | *rs8075846* |  |
| *rs2059606* | *rs8122* |  |
| *rs4129148* | *rs9639436* |  |
| *rs831628* | *rs9882205* |  |
| *rs1929480* | *rs11078855* |  |
| *rs12917707* | *rs11119568* |  |
|  | *rs1184835* |  |
|  | *rs12535365* |  |
|  | *rs12877336* |  |
|  | *rs2683764* |  |
|  | *rs35958249* |  |
|  | *rs4256629* |  |
|  | *rs4571803* |  |
|  | *rs493430* |  |
|  | *rs615204* |  |
|  | *rs693916* |  |
|  | *rs7108425* |  |
|  | *rs7118682* |  |
|  | *rs730275* |  |
|  | *rs9294168* |  |
|  | *rs9690688* |  |

1 *rs7725* primers CAGAAGTTGGAGAAGTGGAGGGATGTG and TAACGGCCAAACTGTTCATCCTAG, restrict with *Tsp*509I, digested allele T, fragment sizes 208 and 69 base pairs; *rs573816* primers GAAGTGCTGTTGGTATGGATTATTTATTG and AAGTGAGATGCAGGAAATCCAAATAG, restrict with *Bst*XI, digested allele C, fragment sizes 113 and 22 base pairs.

**Supplemental Figure 1** Correlation of self-reported number of EP grandparents with estimated EP ancestry using 67 genomic control markers and STRUCTURE software in controls (A: r^2^=0.55) and cases (B: r^2^=0.36).
